# Supplementary material for: Direct glia-to-neuron transdifferentiation gives rise to a pair of male-specific neurons that ensure nimble male mating
Source: eLife. 2020 Nov 3;9:e48361. doi: 10.7554/eLife.48361 (PMC7609048; doi:10.7554/eLife.48361)
Supplement: Transparent reporting form [file elife-48361-transrepform.docx]

***eLife’s* transparent reporting form**

We encourage authors to provide detailed information *within their submission* to facilitate the interpretation and replication of experiments. Authors can upload supporting documentation to indicate the use of appropriate reporting guidelines for health-related research (see [EQUATOR Network](http://www.equator-network.org/%20)), life science research (see the [BioSharing Information Resource](https://biosharing.org/)), or the [ARRIVE guidelines](http://www.plosbiology.org/article/info:doi/10.1371/journal.pbio.1000412) for reporting work involving animal research. Where applicable, authors should refer to any relevant reporting standards documents in this form.

If you have any questions, please consult our Journal Policies and/or contact us: [editorial@elifesciences.org](mailto:editorial@elifesciences.org).

**Sample-size estimation**

- You should state whether an appropriate sample size was computed when the study was being designed
- You should state the statistical method of sample size computation and any required assumptions
- If no explicit power analysis was used, you should describe how you decided what sample (replicate) size (number) to use

Please outline where this information can be found within the submission (e.g., sections or figure legends), or explain why this information doesn’t apply to your submission:

No sample size estimation was computed *a priori* for expression analysis in *C. elegans* as sample limitation is not an issue working with this model organism. We selected minimum sample sizes basing on what is customary in the field and on our previous experience (Sammut et al., 2015; Barrios et al., 2012).

Information about sample size can be found in the Source Data files, as well as in figures and figure legends in the main manuscript.

**Replicates**

- You should report how often each experiment was performed
- You should include a definition of biological versus technical replication
- The data obtained should be provided and sufficient information should be provided to indicate the number of independent biological and/or technical replicates
- If you encountered any outliers, you should describe how these were handled
- Criteria for exclusion/inclusion of data should be clearly stated
- High-throughput sequence data should be uploaded before submission, with a private link for reviewers provided (these are available from both GEO and ArrayExpress)

Please outline where this information can be found within the submission (e.g., sections or figure legends), or explain why this information doesn’t apply to your submission:

Information about the number of replicates can be found in the figures and/or figure legends and Source Data files for each figure.

For reporter expression analysis, mutant analysis, sex-transformation experiments and RNAi knock down experiments, the number of animals tested account for biological replicates. EdU experiments were performed four times. Here, repetitions also account for biological replicates (explained in detail in Source Data 3).

In the case of the behavioural experiments each Source Data file indicates the number of animals tested (biological replicate) and the number of experiments performed (technical replicates).

No exclusions were made except for behavioural experiments. Criteria for inclusion/exclusion of data is clearly stated in the figure legends and in the Materials and Methods section of the main manuscript.

**Statistical reporting**

- Statistical analysis methods should be described and justified
- Raw data should be presented in figures whenever informative to do so (typically when N per group is less than 10)
- For each experiment, you should identify the statistical tests used, exact values of N, definitions of center, methods of multiple test correction, and dispersion and precision measures (e.g., mean, median, SD, SEM, confidence intervals; and, for the major substantive results, a measure of effect size (e.g., Pearson's r, Cohen's d)
- Report exact p-values wherever possible alongside the summary statistics and 95% confidence intervals. These should be reported for all key questions and not only when the p-value is less than 0.05.

Please outline where this information can be found within the submission (e.g., sections or figure legends), or explain why this information doesn’t apply to your submission:

All statistical analysis parameters and results are provided in the Source Data file of each experiment performed. The statistical tests used are also described in each figure legend and annotated on the figures themselves.

(For large datasets, or papers with a very large number of statistical tests, you may upload a single table file with tests, Ns, etc., with reference to sections in the manuscript.)

**Group allocation**

- Indicate how samples were allocated into experimental groups (in the case of clinical studies, please specify allocation to treatment method); if randomization was used, please also state if restricted randomization was applied
- Indicate if masking was used during group allocation, data collection and/or data analysis

Please outline where this information can be found within the submission (e.g., sections or figure legends), or explain why this information doesn’t apply to your submission:

As stated in the Materials and Methods section, all behavioural experiments were analysed blind to the manipulation. Videos of mating recordings were scored blindly by two observers. In the case of calcium imaging analysis, peaks were identified manually by and observer blind to the genotype and treatment.

**Additional data files (“source data”)**

- We encourage you to upload relevant additional data files, such as numerical data that are represented as a graph in a figure, or as a summary table
- Where provided, these should be in the most useful format, and they can be uploaded as “Source data” files linked to a main figure or table
- Include model definition files including the full list of parameters used
- Include code used for data analysis (e.g., R, MatLab)
- Avoid stating that data files are “available upon request”

Please indicate the figures or tables for which source data files have been provided:

A Source Data files have been provided for each of the figures, including figure supplements. with the exception of Figure 1, Figure 2, Figure 3 – figure supplements 1 and 3, Figure 6 – figure supplement 1 and Figure 7 – figure supplement 1.
